# RNA-Seq Analysis of *Cocos nucifera*: Transcriptome Sequencing and *De Novo* Assembly for Subsequent Functional Genomics Approaches

**DOI:** 10.1371/journal.pone.0059997

**Published:** 2013-03-29

**Authors:** Haikuo Fan, Yong Xiao, Yaodong Yang, Wei Xia, Annaliese S. Mason, Zhihui Xia, Fei Qiao, Songlin Zhao, Haoru Tang

**Affiliations:** 1 College of Horticulture, Sichuan Agricultural University, Ya’an, Sichuang province, P.R. China; 2 Hainan Key Laboratory of Tropical Oil Crops Biology/Coconut Research Institute, Chinese Academy of Tropical Agricultural Sciences, Wenchang, Hainan, P.R. China; 3 Tropical Corps Genetic Resources Institute/Key Laboratory of Crop Gene Resources and Germplasm Enhancement in Southern China, Chinese Academy of Tropical Agricultural Sciences, Danzhou, Hainan province, P.R. China; 4 School of Agriculture and Food Sciences and Centre for Integrative Legume Research, The University of Queensland, Brisbane, Australia; 5 Hainan Key Laboratory for Sustainable Utilization of Tropical Bioresources/Institute of BioScience and Technology, College of Agriculture, Hainan University, Haikou, Hainan province, P.R China; University of Georgia, United States of America

## Abstract

**Background:**

*Cocos nucifera* (coconut), a member of the Arecaceae family, is an economically important woody palm grown in tropical regions. Despite its agronomic importance, previous germplasm assessment studies have relied solely on morphological and agronomical traits. Molecular biology techniques have been scarcely used in assessment of genetic resources and for improvement of important agronomic and quality traits in *Cocos nucifera*, mostly due to the absence of available sequence information.

**Methodology/Principal Findings:**

To provide basic information for molecular breeding and further molecular biological analysis in *Cocos nucifera*, we applied RNA-seq technology and *de novo* assembly to gain a global overview of the *Cocos nucifera* transcriptome from mixed tissue samples. Using Illumina sequencing, we obtained 54.9 million short reads and conducted *de novo* assembly to obtain 57,304 unigenes with an average length of 752 base pairs. Sequence comparison between assembled unigenes and released cDNA sequences of *Cocos nucifera* and *Elaeis guineensis* indicated that the assembled sequences were of high quality. Approximately 99.9% of unigenes were novel compared to the released coconut EST sequences. Using BLASTX, 68.2% of unigenes were successfully annotated based on the Genbank non-redundant (Nr) protein database. The annotated unigenes were then further classified using the Gene Ontology (GO), Clusters of Orthologous Groups (COG) and Kyoto Encyclopedia of Genes and Genomes (KEGG) databases.

**Conclusions/Significance:**

Our study provides a large quantity of novel genetic information for *Cocos nucifera*. This information will act as a valuable resource for further molecular genetic studies and breeding in coconut, as well as for isolation and characterization of functional genes involved in different biochemical pathways in this important tropical crop species.

## Introduction

*Cocos nucifera* is an economically important crop species widely grown in tropical and subtropical regions. The coconut tree is highly versatile, with extensive applications in the fields of agriculture and industry: food, fiber, oil, soil fertilizers, spa ingredients, furniture, fashion accessories, garments, construction and building materials, oleochemicals and biofuels. *Cocos nucifera* is generally separated into two types based on morphological characteristics: tall and dwarf coconut. The types differ in life span, with 4–6 years required for inflorescence production in dwarf coconut and 8–10 years in tall coconut [1]. This long life-cycle is a major limiting factor in improving the yield and quality traits of *Cocos nucifera* through conventional breeding approaches. The discovery of novel genes and the development of molecular markers linked to quality and agronomic traits may speed up the pace in C*ocos nucifera* breeding. However, it is difficult to isolate functional genes which govern important quality and agronomic traits in coconut due to the scarcity of available genetic sequences. Currently, there are only 774 sequences available in the National Center for Biotechnology Information (NCBI) database originating from *Cocos nucifera*.

Transcriptome sequencing is an efficient methodology for large scale gene discovery [2]. However, transcript sequencing was previously primarily dependent on the construction of clone libraries [3]. With the development of new sequencing technologies, direct sequencing of cDNA fragments can now be accomplished free of cloning. Compared with earlier cloning methods, massively parallel sequencing of RNA (RNA-Seq) has a dramatically increased RNA sequencing output, as well as allowing global measurement of transcript abundance [4], [5]. Hence, RNA-Seq is a positive driving force for characterizing the transcriptome and elucidating transcriptome complexity. Today, RNA-Seq is performed based on next-generation sequencing technology to generate millions of short cDNA reads. Following this, two strategies can be applied to construct transcripts using these short reads. The first strategy, proposed by Trapnell et al. [6] and Guttman et al. [7], is an ‘align-then-assemble’ approach: based on this method, the transcript can be reconstructed by aligning the short reads to the genome and then accounting for possible splice events. The second strategy is called ‘assemble-then-align’: *de novo* assembly is used to construct transcripts, and then the assembled transcripts are aligned to the genome to elucidate intron and exon structure and variations between alternatively spliced transcripts [8], [9]. Moreover, *de novo* assembly is also applied to construct the transcriptome for these no-model organisms without genome sequences. In order to perform *de novo* assembly, very abundant short reads are required. RNA-seq has enhanced our understanding of transcriptomes in the animal and plant kingdoms, revealing novel transcriptome sequences, alternative splicing, transcript isoforms, new large intergenic noncoding RNAs and single nucleotide polymorphisms (SNPs) [10], [11]. RNA-seq analysis provides a promising avenue for capturing genome-wide transcripts and splicing in unprecedented detail.

In this study, we used Illumina RNA-Seq technology to generate 54,931,406 short reads containing a total of 4,943,826,540 nucleotide bases. Due to the scarcity of reference sequences available in the National Center for Biotechnology Information (NCBI) and other databases, *de novo* assembly was applied to combine these short reads: 57,304 unigenes were obtained with an average length of 752 bp. These unigenes were aligned with sequence databases for subsequent Blast, mapping and annotation analyses. These results will provide a platform of sequence information for global discovery of novel functional genes in *Cocos nucifera*.

## Materials and Methods

### Plant Materials and RNA Isolation

Spear leaves, young leaves and fruit flesh were sampled from Hainan Tall cultivars, for total RNA isolation. High quality RNA was obtained using the MRIP (Methods for RNA Isolation from Palms) protocol [12], which contains an extraction buffer based on that proposed by Bilgin et al. [13], but modified for suitability in Palmaceae RNA extractions. RNA integrity was confirmed using agarose gel electrophoresis and a Agilent 2100 Bioanalyzer (Agilent Technologies). Isolated RNAs from different tissues were mixed for further processing. Beads with dT oligos were used to isolate poly (A) mRNA from the total RNA (Qiagen GmbH, Hilden, Germany).

### Synthesis of cDNA and Subsequent Sequencing

The purified mRNA was fragmented with divalent cations under increased temperature. These short fragments were taken as templates to synthesize the first-strand cDNA using random hexamer primers and superscript^TM^III (Invitrogen™, Carlsbad, CA, USA). Second-strand cDNA was then synthesized in a solution containing buffer, dNTP, RNaseH and DNA polymerase I and subsequently purified using a QiaQuick PCR extraction kit (Qiangen). EB buffer was used to resolve these short fragments for end reparation and poly (A) addition. The sequence adaptors were linked to two ends of short cDNA sequences and suitably sized cDNA fragments were selected out for PCR amplification based on the agrose gel electrophoresis results. Finally, the library established was sequenced with an Illumina Hiseq™ 2000. The paired-end library was developed according to the protocol of the Paired-End sample Preparation kit (Illumina, USA).

### De novo Assembly

Transcriptome *de novo* assembly was carried out using the short read assembly program “Trinity”, following the protocol documented in Grabherr et al. [14] and Xiao et al. [12].

### Annotation and Classification of Unigenes

Unigenes were used for BLAST searches and annotation against the NCBI Nr database (NCBI non-redundant sequence database) using an E-value cut-off of 10^−5^ (E-value <0.00001). Unigene sequences were also aligned by BLASTX to protein databases such as Swiss-Prot, KEGG and COG, in order to retrieve proteins with the highest sequence similarity to the given unigenes along with putative functional annotations. If results of different databases conflicted, Nr then Swiss-prot database results were given precedence. For unigenes that did not align to any of the above databases, ESTScan software [15] was used to predict their coding regions and determine sequence direction. Unigenes aligned to databases with higher priority will not be aligned to lower priority database. The alignments were considered complete when all four assignments were finished. Coding sequence regions were then determined for the highest-ranked proteins using BLAST. Unigenes that could not be aligned to any database were scanned by ESTScan [15] to determine the nucleotide (5′–3′) and amino acid sequences of the coding regions.

The Blast2GO program was used to obtain GO annotations for the unigenes, as well as for the KEGG and COG analyses [16]. The WEGO software was then used to perform GO functional classification of all unigenes, in order to view the distribution of gene functions within coconut at the macro level [17]. This analysis mapped all of the annotated unigenes to GO terms in the database and calculated the number of unigenes associated with every GO term. COG and KEGG pathway annotations were performed using Blastall software against the COG and KEGG databases.

### Alignment with Available Cocos Nucifera and Elaeis Guineensis EST Sequences and the Phoenix Dactylifera Genome

A total of 115 EST sequences for *Cocos nucifera*, 41,977 EST sequences for related specie *Elaeis guineensis* and the *Phoenix dactylifera* genome were downloaded from NCBI database. These sequences were then used for nucleotide Blast searches against the 57,304 unigenes, based on an E-value threshold of 10^−5^ (E-value <0.00001).

## Results

### Sequencing and Assembly Quality Statistics

To globally elucidate the transcriptome of *Cocos nucifera*, RNA samples from different tissues were mixed in equal proportions and then used for mRNA preparation, fragmentation and cDNA synthesis. Two independent Illumina sequencing runs generated a total of 54,931 406 short sequence reads consisting of 4,943,826,540 nucleotides (nt) total, with an average length of 90 bp for each short read (Table 1).

**Table 1:** Summary of RNA-Seq and *de novo* assembly of *Cocos nucifera* results.

	Number	Mean size	N50 size	Total nucleotides
Read	54,931,406	90	90	4,943,826,540
Contig	127,952	344	594	43,994,141
Unigene	57,304	752	1,219	43,090,665

**Figure 1:**
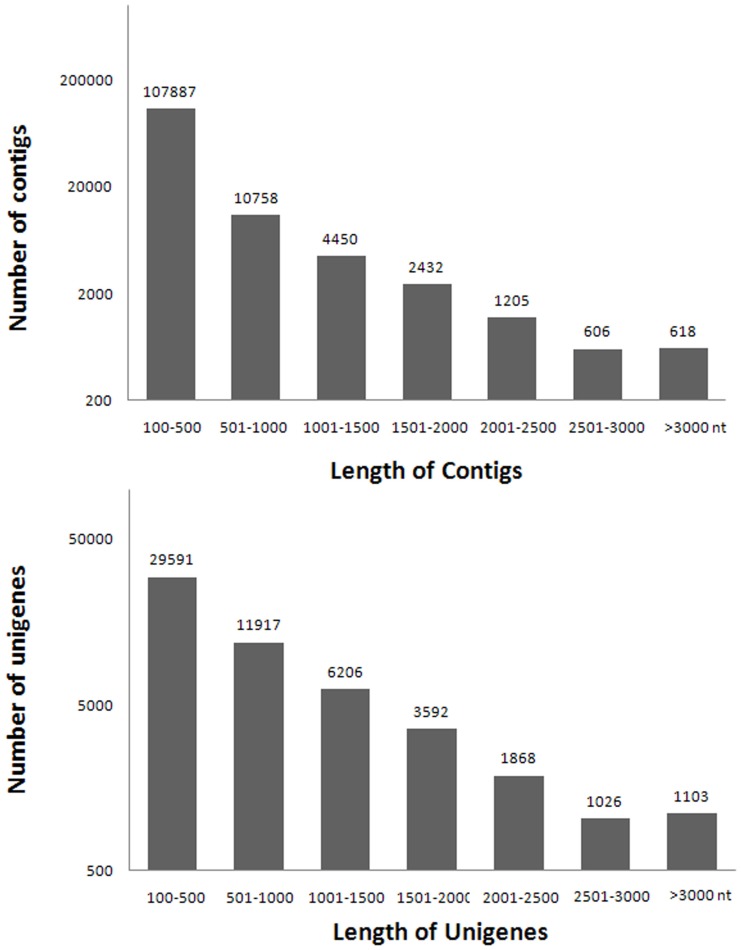
Statistical analysis of a *de novo* assembly of *Cocos nucifera* short reads generated by Illumina Hiseq™ 2000 sequencing. The distribution of assembled contigs and unigene lengths is shown (127,952 contigs and 57,304 unigenes were generated in the study).

Due to the absence of reference genomic sequences, *de novo* assembly was applied to construct transcripts from these RNA-seq reads. *De novo* assembly was performed using Trinity (insert reference or company here), a *de novo* assembler of RNA-seq. 127,952 contigs were constructed from the raw sequence reads. As shown in Figure 1A, the sequence length of these assembled contigs ranged from 100 bp to more than 3000 bp, with an average length of 344 bp. The number of contigs decreased with increasing contig length (Table 1, Figure 1A).

Using paired-end reads, contigs from the same transcript were connected and extended in both ends to get the longest unigenes possible. In total 57,304 unigenes were generated, with an average unigene length of 752 bp and ranging in size from 200 bp to more than 3000 bp (Table 1 and figure 1B). These assembled unigenes have been deposited in the NCBI database.

### Comparison of Assembled Unigenes with EST Sequences from Cocos Nucifera and Elaeis Guineensis Available in the NCBI Database

**Figure 2:**
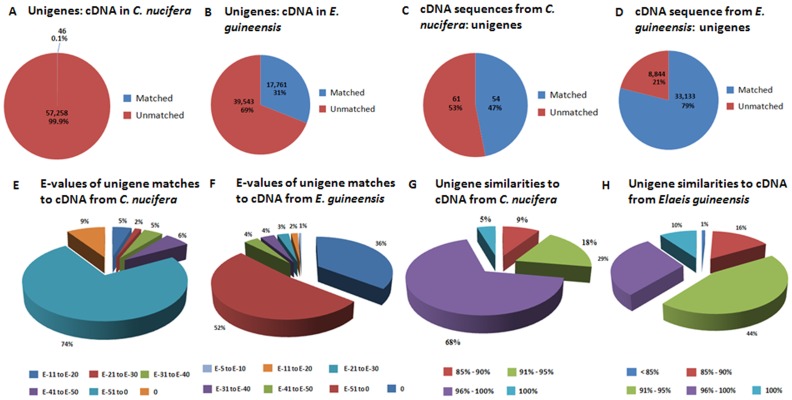
Summary of the nucleotide BLAST results between the *de novo*-assembled unigenes and EST sequences from *Cocos nucifera* and *Elaeis guineensis*. (A) Percentage of unigenes matched with EST sequences from *Cocos nucifera*. (B) Percentage of unigenes matched with EST sequences from *Elaeis guineensis*. (C) Percentage of EST sequences from *Cocos nucifera* matched with unigenes. (D) Percentage of EST sequences from *Elaeis guineensis* matched with unigenes. (E) E-values of unigene matches with cDNA sequences from *Cocos nucifera*. (F) E-values of unigene matches with cDNA sequences from *Elaeis guineensis*. (G) Unigene similarities to cDNA sequences from *Cocos nucifera*. (H) Unigene similarities to cDNA sequences from *Elaeis guineensis.*

To estimate the accuracy of the *de novo* assembly performed in the research, the assembled sequences (57 304 unigenes) were blasted against available *Cocos nucifera* cDNA sequences downloaded from the NCBI database in February 2012. These comprised 115 ESTs (JG390663-JG390776, AF241736, AM259062-AM259066) generated from water stressed plantlets. Due to the limited number of EST sequences available for *Cocos nucifera*, all available EST sequences from related species *Elaeis guineensis* (41,977 reads) were also downloaded from the NCBI database for sequence alignments with the 57,304 unigenes. Using a cut-off E-value of 10^−5^, 46 unigenes (0.1%) could be aligned to the downloaded *Cocos nucifera* cDNA sequences (Figure 2A), and 31% of the unigenes could be aligned to the *Elaeis guineensis* cDNA sequences (Figure 2B). Only 47% of the NCBI EST sequences (all from water-stressed plantlets) of *Cocos nucifera* could be matched with unigenes (Figure 2C), but 79% of the EST sequences from *Elaeis guineensis* could be matched with unigenes (Figure 2D).

A high degree of similarity between the assembled unigenes and the available EST sequences of *Cocos nucifera* and *Elaeis guineensis* was detected. In *Cocos nucifera* and *Elaeis guineensis*, 83% and 88% of the matched sequences respectively had an E-value lower than 10^−51^ (Figure 2E, F), and 91% and 83% had alignment identities higher than 90%, supporting the reliability of the *de novo* assembly performed in the study (Figure 2G, H).

In order to estimate the coverage of the coconut transcriptome over all genes in the species, we downloaded the whole genome sequence of *Phoenix dactylifera* (a relative of *Cocos nucifera*) from the website (http://qatar-weill.cornell.edu/research/datapalmGenome/download.html). There are 28,889 genes predicted in the *Phoenix dactylifera* genome [18]. Using a cut-off E-value of 10^−5^, 20,541 (71.1%) of genes predicted from the genome sequence matched to the coconut transcriptome (Table S1), which suggests that the coconut transcriptome may have high coverage over all genes in the genome.

### Annotation and Classification of Cocos Nnucifera Unigenes

To annotate the *Cocos nucifera* transcriptome, unigene sequences were first searched against the NCBI non-redundant (Nr) protein database using a cut-off E-value of 10^−5^. Out of 57,304 unigenes, 39 109 unigenes (68%) were matched to known protein sequences in the Nr database (Table 2), while 18,195 (32%) unigenes could not be aligned to the Nr database. Out of 562,992 hits, 207 960 (37%) (representing 17,849 unigenes - 31%) had an E value <1e−50. When aligned to the four model plant protein databases *Arabidopsis thaliana* (model system for dicot plants) (Table 3), *Zea mays*, *Oryza sativa* (model system for monocot plants) and *Arabidopsis lyrata*, *Cocos nucifera* unigenes had the highest identities with the *Oryza sativa* protein database (Table 3). At a cut-off E-value of 1e^−5^, 34,708 (61%) of *Cocos nucifera* unigenes had matches to the *Oryza sativa* protein database, 59% to the *Arabidopsis thaliana* database, 34% to the *Zea mays* database and 30% to the *Arabidopsis lyrata* database. We also found that 17,886 (31%) of unigenes had matches to the *Oryza sativa* protein database with an E-value <1e^−50^. At this E-value 22%, 16% and 14% of unigenes matched to the *Arabidopsis thaliana*, *Zea mays* and *Arabidopsis lyrata* databases respectively.

**Table 2:** Summary of annotations of assembled *Cocos nucifera* unigenes.

Category	Account	Percentage (%)^b^
Nr^a^ annotated unigenes	39,109	68.2%
Unique Nr proteins	29,425	51.3%
Swissprot	25,293	44.1%
GO classified unigenes	15,178	26.5%
COG classified unigenes	14,741	25.7%
KEGG classified unigenes	23,128	40.4%

a Nr: NCBI non-redundant sequence database.

b Percentage of annotated unigenes in total 57,307 assembled unigenes of coconut.

**Table 3:** Statistical analysis of *Cocos nucifera* unigenes with sequence matches against public protein databases.

Database	E–value range	No. of matched Unigenes	No. of matched proteins in Nr
*Arabidopsis thaliana*	(0–1e^−5^)	33,120 (59%)^b^	145,825 (32%)^c^
(457 052)^a^	(1e^−10^–1e^−5^)	4,149 (7%)	18,695 (4%)
	(1e^−50^–9e^−11^)	16,122 (28%)	71,257 (16%)
	(0–1e^−51^)	12,851 (22%)	55,872 (12%)
*Zea mays*	(0–1e^−5^)	19,643 (34%)	45,620 (38%)
(119 770)	(1e^−10^–1e^−5^)	2,915 (5%)	4,945 (4%)
	(1e^−50^–9e^−11^)	9,968 (17%)	21,559 (18%)
	(0–1e^−51^)	9,239 (16%)	19 117 (16%)
*Oryza sativa*	(0–1e^−5^)	34,708 (61%)	122,467 (44%)
(279 003)	(1e^−10^–1e^−5^)	9,571 (17%)	17,540 (6%)
	(1e^−50^–9e^−11^)	23,888 (42%)	61,620 (22%)
	(0–1e^−51^)	17,886 (31%)	43,307 (16%)
*Arabidopsis lyrata*	(0–1e^−5^)	17,430 (30%)	33,512 (45%)
(74 331)	(1e^−10^–1e^−5^)	2,629 (5%)	3,882 (5%)
	(1e^−50^–9e^−11^)	9,248 (16%)	16,511 (22%)
	(0–1e^−−51^)	7,783 (14%)	13,120 (18%)

a Total protein sequences of the corresponding species from Nr database.

b Percentage of matched sequences in total unigenes of coconut.

c Percentage of matched sequences in total protein sequences used in this species.

**Figure 3:**
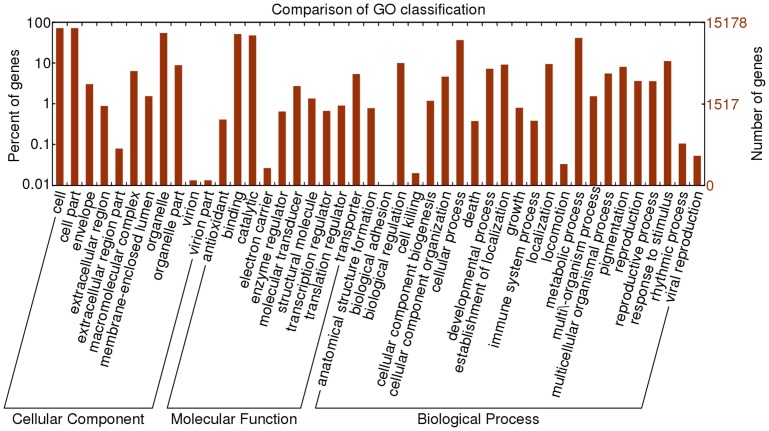
Histogram of GO classifications of assembled *Cocos nucifera* unigenes. Results are summarized for three main GO categories: biological process, cellular component and molecular function.

**Figure 4:**
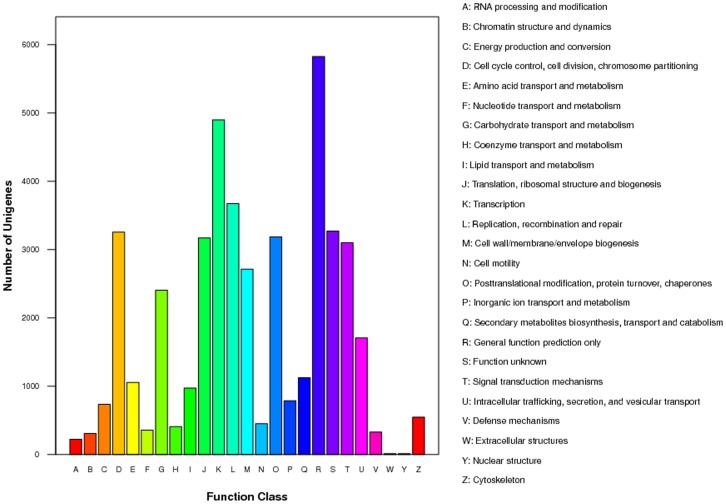
Histogram of cluster of COG classification of *Cocos nucifera* sequences deposited in the NCBI database and *de novo* assembled unigenes. 14,741 assembled unigenes were annotated, falling into 25 clusters.

Gene Ontology (GO) terms were subsequently assigned to *Cocos nucifera* unigenes based on their sequence matches to known protein sequences in the Nr database. A total of 15,178 unigenes (26.50%) were assigned at least one GO term (Table 2), amongst which 8,022 (14.00%) were in the biological process category, 10,986 (19.17%) were in the cellular component category and 9,532 (16.63%) were in the molecular function category. Based on GO annotation, *Cocos nucifera* unigenes were categorized into 44 groups using a set of plant-specific GO categories. Metabolic processes (5,538 unigenes, 9.66%), cell (10,874 unigenes, 18.98%) and catalytic activity (6,531 unigenes, 11.40%) were the most abundant GO slims in each of the biological processes, cellular component localization and molecular functionality categories respectively (Figure 3). The high number of unigenes putatively involved in metabolic processes (5,538) and cellular processes (5,285) in the biological processes category indicated that the *Cocos nucifera* tissues used in the study were undergoing extensive metabolic activities. Also, a large number of expressed genes were involved in biological regulation (1,522), developmental processes (1,087), establishment of location (1,341), location (1,485), response to stimulus (1,751), cell part (9,905), organelle (8,265), and binding (6,090), while only a few unigenes were clustered in terms of adhesion, cell killing, locomotion, nitrogen utilization, pigmentation, rhythmic process, viral reproduction, cell junction, extracellular region part, virion, and antioxidant activity. Figure 4 shows the GO classification of coconut transcriptome from RNA mixture of leaves and fruits.

In order to further elucidate the functionality of the *Cocos nucifera* transcriptome, the annotated unigenes were categorized into different functional groups based on the COG database (Cluster of Orthologus Groups), (Figure 4). Out of 57,304 unigenes, 14,741 could be classified into 25 COG categories. Out of 14,741 unigenes (Table 2), 5,825 (39.52%) were assigned into the COG category of general function prediction, which represented the largest functional group of the 25 COG categories, followed by transcription (4,899, 33.23%), replication, recombination and repair (3,674, 24.92%), unknown function (3,270, 22.18%), cell cycle control, cell division and chromosome partitioning (3,256, 22.09%), signal transduction mechanisms (3,099, 21.02%), cell wall/membrane/envelope biogenesis (2,714, 18.41%), carbohydrate transport and metabolism (2,404, 16.31%), intracellular trafficking, secretion, and vesicular transporters (1,706, 11.57%), and secondary metabolite biosynthesis, transport and catabolism (1,124, 7.62%). The two categories involving extracellular structures and nuclear structure each consisted of 10 unigenes (0.07%), representing the smallest COG classifications. In addition, small numbers of unigenes from the functional classes of lipid transport and metabolism (971 unigenes), cell motility (449 unigenes), inorganic ion transport and metabolism (787 unigenes), defense mechanisms (327 unigenes) and cytoskeleton (546 unigenes) were also identified.

To further identify the active biochemical pathways in the leaves and fruit of *Cocos nucifera*, we mapped the *Cocos nucifera* unigenes to the reference canonical pathways in the Kyoto Encyclopedia of Genes and Genomes (KEGG). KEGG is thought to provide a basic platform for systematic analysis of gene function in terms of the network of gene products. A total of 23,168 unigenes were annotated based on a BLASTX search of the KEGG database (Table 2): 215 biosynthesis pathways were predicted. Of these 215 KEGG pathways, the metabolic pathway was the largest, containing 6,356 members (unigene products). Other pathways included endocytosis (2,686 members), glycerophospholipid (2,623 members), ether lipid metabolism (2,489 members), biosynthesis of secondary metabolites (1,829 memebers), RNA transport (1,583 members), plant-pathogen interaction (1,316 members), plant hormone signal transduction (1,287 members), mRNA surveillance pathway (1,111 members) and spliceosome (1,003 members).

### Genes Potentially Involved in Fatty Acid Biosynthesis and Metabolism

As well as being a fruit and food crop, *Cocos nucifera* is an important tropical oil crop. The oil content of coconut flesh is generally between 64% and 75% dry weight, of which more than 50% is lauric acid. Hence, we further characterized the assembled unigenes with regards to biochemical pathways related to fatty acid biosynthesis and metabolism: 347 unigenes were classified as being involved in five steps of the fatty acid biosynthesis and metabolism pathway (fatty acid biosynthesis, unsaturated fatty acid, citrate cycle, fatty acid metabolism and fatty acid elongation) (Table 4). Of the 347 unigenes, 121 were involved in fatty acid biosynthesis, and then followed by fatty acid metabolism (94), fatty acid biosynthesis (81), unsaturated fatty acid (41) and fatty acid elongation.

**Table 4:** Classification of 347 unigenes involved in fatty acid biosynthesis and metabolism.

Pathway	Genenumber	Types of Genes
Fatty acid biosysthsis	81	acetyl CoA:ACP carboxylase (21)^a^
		beta-ketoacyl-ACP synthase (22)
		beta-ketoacyl-ACP reductase (13)
		enoyl-ACP reductase (4)
		fatty acyl-ACP thioesterase (20)
		beta-hydroxyacyl-ACP dehydratase (1)
Unsaturated fatty acid	41	Oxidoreductases (14)
		fatty acid desaturase (9)
		cytochrome P450 (9)
		stearyl-ACP desaturase (9)
Citrate cycle	127	citrate synthase (17)
		malate dehydrogenase (20)
		isocitrate dehydrogenase (21)
		pyruvate dehydrogenase (23)
		oxoglutarate dehydrogenase (16)
		succinate dehydrogenase (8)
		phosphoenolpyruvate carboxykinase (10)
		fumarate hydratase (1)
		Acon5itate hydratase (8)
		succinate-CoA ligase (3)
Fatty acid metabolism	94	long-chain-fatty-acid-CoA synthetase (25)
		acyl-CoA oxidase (16)
		acetyl-CoA C-acetyltransferase (5)
		acetyl-CoA C-acyltransferase (7)
		acyl-CoA dehydrogenase (1)
		alcohol dehydrogenase (26)
		aldehyde dehydrogenase(8)
		enoyl-CoA hydratase (6)
Fatty acid elongation	4	quinone reductase (1)
		trans-2-enoyl-CoA reductase (2)
		palmitoyl-protein thioesterase (1)

a Number of unigenes with annotated functions.

### Prediction of Non-coding RNA in Cocos Nucifera

To predict non-coding RNA in the coconut transcriptome, 57,304 uingenes were blasted against the *Phoenix dactylifera* (relative of *Cocos nucifera*) whole genome sequence. The alignment results showed that 4,433/57,304 unigenes (7.7%) had high identity (E-value <10^−50^) with intergenic sequences of *Phoenix dactylifera*, suggesting that these expressed unigenes may comprise non-coding RNA (Table S2). Sequence length of the putative non-coding RNA ranged from 114 bp to 1004 bp. For further characterisation of these putative non-coding RNAs, the 4,433 unigenes were aligned to the Nt, miRBase and lncRNAdb databases. Using a cut-off E-value of 10^−5^, only 10 unigenes could be classified as containing known non-coding RNA. Three different types of non-coding RNA were predicted: seven unigenes (unigene 2783, unigene 37389, unigene 41901, unigene 52461, unigene 54261, unigene 54262, and unigene 7600) contained matches to primary miRNA sequences in the miRBase database; unigene41380 matched to 18S rRNA and two unigenes (unigene 51756 and unigene 7052) had matches to tRNA sequences. However, due to scarcity of sequence information for non-coding RNAs in the plant databases, the remaining 4,423 unigenes could not be characterized.

## Discussion

Coconut palms can provide almost all the necessities of life in the tropical regions, such as food, drink, oil, etc. However, due to the long life cycle of the coconut palm and the lack of genetic sequence information, efforts to improve the agronomic characteristics of *Cocos nucifera* through molecular breeding have made little progress. Transcriptome sequencing can provide abundant sequence information, as well as shedding some light on the basic biological processes occurring in cells [19], [20]. With the advent of next-generation sequencing technology, transcriptome analysis has been widely applied to many different species [21], [22]. However, there is still no available transcriptome sequencing data for coconut. In this study, we utilized RNA-seq technology to sequence the coconut transcriptome, and discovered a large number of novel genes in *Cocos nucifera*. A total of 57,304 unigenes were obtained, representing a comprehensive transcriptome of *Cocos nucifera*. Functional annotation showed that these unigenes covered every basic biological process, and 23,168 of these unigenes were also mapped into 215 KEGG pathways. We provide comprehensive sequence resources for coconut for the first time, which will benefit subsequent functional genomics and discovery of novel genes in *Cocos nucifera*.

In this study, Illumina sequencing produced 54,931,406 short reads (average 90 bp each short read), totaling 4.9 Gb of sequence. Generally, there are two alternative computational methodologies for establishing transcriptomes based on these short reads: the mapping-first approach, which is carried out based on an available reference genome, and the assembly-first (*de novo*) approach. Due to absence of reference sequences for *Cocos nucifera*, *de novo* assembly was applied to connect these short reads to form longer expressed sequences. A number of assembly software programs are available for this purpose, such as ABySS [23], SOAPdenovo [24], [25] and Oases [26]. Previous whole transcriptome assemblies in plants have been done using SOAPdenovo software, a whole-genome assembler with utility for assembling short reads generated by Illumina sequencing. The accuracy of the *de novo* assembly performed using SOAPdenovo software is strongly dependent on the user-defined parameter of overlap sequence length (k-mer), meaning the overlap required between two short reads in order to consider them contiguous. Wang et al. [27] used 21-mers to assemble the transcriptome of whitefly, and obtained an average unigenes length of 226 bp using this parameter. When k-mer = 23 was used to connect short sequence reads generated by Illumina in another study, 74,336 assembled transcript sequences were obtained, with an average of 439.5 bp in sequence length. Xia et al. [28] applied a more rigorous k-mer parameter (k = 29) to assemble trascriptome fragments, and obtained an average unigenes length of 436 bp. In our research, *de novo* assembly was carried out by using Trinity, a *de novo* assembler of RNA-seq. Compared to other *de novo* assemblers of RNA-seq, Trinity software is thought to be more suitable for constructing a *de novo* transcriptome without a reference genome, as it utilizes the kmer graph method to assemble Illumina RNA-seq reads [14]. Using Trinity software, 54,931,406 short reads were assembled into contigs, which were subsequently connected into unigenes. The average sequence length of the unigenes was 752 bp, which is longer than previously documented sequence lengths using other *de novo* assembler programs [29], [30], [31]. In addition, the sequence length of 13,795 (24.1%) unigenes in our study was more than 1000 bp. Hence, our work provides some full-length RNA-seq data (putative complete genes), which will be advantageous for further gene function validation and functional genomics approaches in *Cocos nucifera*.

It is essential to evaluate whether unigene assemblies are reliable, particularly in *de novo* sequencing. Xia et al. [28] assembled Illumina short reads of *Hevea brasiliensis*, and matched the assembled unigenes to known *H. brasiliensis* cDNA sequences to confirm the accuracy of the *de novo assembly*: 31% of matches had an E-value lower than 10^−50^. In our study, due to the scarcity of available cDNA sequences from *Cocos nucifera*, the assembled unigenes were separately matched with EST sequences from both *Cocos nucifera* and *Elaeis guineensis*: 83% and 88% of matched sequences for *Cocos nucifera* and *Elaeis guineensis* respectively had an E-value lower than 10^−51^. This high degree of sequence match similarity indicated that the *de novo* assembly we performed using the Trinity software was very accurate.

Out of 57,304 unigenes, 39,109 (68.2%) were matched against the NCBI non-redundant (Nr) protein database, allowing further functional annotation and classification using GO, COG and KEGG. This functional annotation provided predicted information for biological function and biosynthesis pathways for the assembled unigenes [32], [33]. Although functional annotation and classification provide predicted functions for these coconut unigenes, ongoing studies are still required for further function validation. Meanwhile, we found that 34,708 (60.57%) of *Cocos nucifera* unigenes had matches in the *Oryza sativa* protein database, which is the highest level of similarity across all the model plant databases. This result is in good accordance with the sequence alignment between rice and data palm, another Palmaceae species, made by Al-Dous [18]. This suggests the *Cocos nucifera* genome is more closely related to the *Oryza sativa* genome than to other model plant genomes. Thus, the sequence database of *Oryza sativa* should be considered as a reference for molecular biology research in *Cocos nucifera*.

Coconut is an important tropical oil crop, providing edible fatty acid for millions of people. Compared with other oil-crops, coconut oil contains a large proportion of lauric acid (more than 50%), a twelve carbon saturated fatty acid which is almost undetectable in rapeseed, peanut and soybean. Previous research has investigated phenotypic variation for oil content and fatty acid composition in *Cocos nucifera* [34], [35]. However, the molecular basis of lauric acid accumulation and its regulation are still unclear. In this study, we identified 347 unigenes involved in the biosynthesis and metabolism of fatty acid. These unigenes could be assigned to five steps of the fatty acid biosynthesis pathway, providing a means of elucidating the molecular mechanisms for fatty acid biosynthesis in coconut palm. It is noteworthy that 20 unigenes were predicted to be related to fatty acyl-ACP thioesterase, which is a crucial enzyme for terminating the elongation of carbon chains and therefore regulating the length of fatty acids. Therefore, it is possible that the expression of fatty acyl-ACP thioesterase is correlated with the observed accumulation of medium chain fatty acids (i.e. lauric acid) in *Cocos nucifera*.

Non-coding RNA (ncRNA) genes can produce functional RNA molecules, rather than encoding proteins [36]. With the technological breakthrough of deep-sequencing, large-scale genome sequencing combined with RNA-seq studies have led to the discovery of numerous non-coding RNAs (ncRNA). Recently, ncRNA has been shown to act in transcriptional and posttranscriptional regulation [37]. In addition to the regulation of gene expression, ncRNA may also play a positive role in maintaining genome stability [38]. Due to the lack of available genome sequence in coconut, we compared the coconut transcriptome to the whole genome sequence of date palm (a species related to coconut). At an E-value cut-off 10^−50^, 4,433 unigenes have matches to the intergenic sequences of the date palm genome. Hence, some unigenes are putative candidates for non-coding RNA in *Cocos nucifera*. However, only 10 unigenes of the 4,423 matching to intergenic sequences could be matched to plant databases of non-coding RNA. Subsequent studies would be required to validate the putative non-coding RNA and to ascertain the biological functionality of these RNA sequences.

### Conclusions

We applied Illumina next-generation sequencing and *de novo* assembly to elucidate the coconut transcriptome for the first time. In total, 57,304 unigenes were obtained, with an average unigene length of 752 bp. Of these unigenes, 99.9% were novel compared to released coconut EST sequences. Of the 57,304 unigenes, 23,168 were mapped into 215 KEGG pathways, including galactose metabolism, plant-pathogen interaction and plant hormone signal transduction pathways. In addition, we also found that 347 unigenes were involved in fatty acid synthesis and metabolism. These unigenes were classified into five steps of the fatty acid biosynthesis and metabolism pathway (fatty acid biosynthesis, unsaturated fatty acid, citrate cycle, fatty acid metabolism and fatty acid elongation). The computational prediction of unigene function will provide primary information for genes involved in different biological processes, allowing these unigenes to be treated as candidate genes in future research.

Overall it can be concluded that the RNA-seq analysis and *de novo* assembly provided a global view of the *Cocos nucifera* transcriptome. The availability of this annotated transcriptome will be a step forward for the isolation and characterization of functional genes involved in different biochemical pathways, as well as paving the way for molecular genetic approaches to *Cocos nucifera* breeding.

## Supporting Information

Table S1 BlastX results between unigenes of *Cocos nucifera* and protein sequence of *Phoenix dactyltfera*.
(XLSX)

Table S2 Alignment results between unigenes and intergenic sequences of *Phoenix dactyltfera*.
(XLSX)
